# Poly(hydrophobic Amino Acids) and Liposomes for Delivery of Vaccine against Group A Streptococcus

**DOI:** 10.3390/vaccines10081212

**Published:** 2022-07-29

**Authors:** Armira Azuar, Harrison Y. R. Madge, Jennifer C. Boer, Jazmina L. Gonzalez Cruz, Jingwen Wang, Zeinab G. Khalil, Cyril Deceneux, Georgia Goodchild, Jieru Yang, Prashamsa Koirala, Waleed M. Hussein, Robert J. Capon, Magdalena Plebanski, Istvan Toth, Mariusz Skwarczynski

**Affiliations:** 1 School of Chemistry and Molecular Biosciences, The University of Queensland, St. Lucia, QLD 4072, Australia; armira.azuar@uq.net.au (A.A.); harrison.madge@uq.net.au (H.Y.R.M.); jingwen.wang1@uq.edu.au (J.W.); jieru.yang@uq.edu.au (J.Y.); p.koirala@uq.edu.au (P.K.); w.hussein@uq.edu.au (W.M.H.); i.toth@uq.edu.au (I.T.); 2 School of Health and Biomedical Sciences, RMIT University, Bundoora West, VIC 3083, Australia; jennifer.boer@rmit.edu.au (J.C.B.); cyril.deceneux@rmit.edu.au (C.D.); georgia.goodchild@rmit.edu.au (G.G.); magdalena.plebanski@rmit.edu.au (M.P.); 3 Diamantina Institute, Faculty of Medicine, The University of Queensland, Woolloongabba, QLD 4102, Australia; j.gonzalezcruz@uq.edu.au; 4 Institute of Molecular Bioscience, The University of Queensland, St. Lucia, QLD 4072, Australia; z.khalil@uq.edu.au (Z.G.K.); r.capon@imb.uq.edu.au (R.J.C.); 5 School of Pharmacy, The University of Queensland, Woolloongabba, QLD 4102, Australia

**Keywords:** peptide-based vaccine, adjuvant, poly(hydrophobic amino acid), liposome, Group A Streptococcus, chain-like nanoparticles

## Abstract

Adjuvants and delivery systems are essential components of vaccines to increase immunogenicity against target antigens, particularly for peptide epitopes (poor immunogens). Emulsions, nanoparticles, and liposomes are commonly used as a delivery system for peptide-based vaccines. A Poly(hydrophobic amino acids) delivery system was previously conjugated to Group A Streptococcus (GAS)-derived peptide epitopes, allowing the conjugates to self-assemble into nanoparticles with self adjuvanting ability. Their hydrophobic amino acid tail also serves as an anchoring moiety for the peptide epitope, enabling it to be integrated into the liposome bilayer, to further boost the immunological responses. Polyleucine-based conjugates were anchored to cationic liposomes using the film hydration method and administered to mice subcutaneously. The polyleucine-peptide conjugate, its liposomal formulation, and simple liposomal encapsulation of GAS peptide epitope induced mucosal (saliva IgG) and systemic (serum IgG, IgG1 and IgG2c) immunity in mice. Polyleucine acted as a potent liposome anchoring portion, which stimulated the production of highly opsonic antibodies. The absence of polyleucine in the liposomal formulation (encapsulated GAS peptide) induced high levels of antibody titers, but with poor opsonic ability against GAS bacteria. However, the liposomal formulation of the conjugated vaccine was no more effective than conjugates alone self-assembled into nanoparticles.

## 1. Introduction

The shift in vaccination approach over the last decade, from conventional whole organism vaccines (e.g., attenuated, inactivated pathogens) to minimalistic subunit vaccines (e.g., carbohydrate-, protein-, or peptide-based antigens) has benefited substantially from the discovery and development of vaccine adjuvants [1,2,3,4,5]. “Adjuvants” can be broadly defined as immunostimulatory components, administered with a vaccine, to enhance the immunogenicity (antigen-specific response) of a vaccine antigen. As a result, adjuvants are key components of most modern vaccines, especially those based on peptide antigens, which are typically poorly immunogenic on their own. In the context of their mechanism of action, adjuvants can be divided into two main types: (a) immune stimulators (classical adjuvants), which directly activate immune cells via receptors, such as Toll-like receptors (TLRs), present on antigen presenting cells (APCs) and (b) delivery systems that protect antigens from degradation while transporting them to lymphatic tissues [1,6]. Not only do particulate delivery systems (such as emulsions, nanoparticles and liposomes) protect antigens from degradation and deliver them to immune cells, they also target antigens to APCs to help induce stronger immune responses [7,8,9,10,11].

Liposomes are artificial phospholipid-bilayer vesicles with an interior aqueous compartment, which have demonstrated excellent ability to deliver therapeutics and vaccines [12,13,14,15,16]. Liposomes have been used as vaccine delivery vehicles since 1974 and a large number of liposome-based products have already reached the market, including vaccines against hepatitis A (Epaxal^®^; Berna Biotech Ltd., formerly Swiss Serum and Vaccine Institute, Bern, Switzerland [17]), influenza (Inflexal^®^ V; Berna Biotech Ltd., Bern, Switzerland [18] and Nasalflu^®^; Berna Biotech Ltd., Bern, Switzerland [19]), malaria (Mosquirix^®^; GSK plc, formerly GlaxoSmithKline plc, London, UK [20]), and shingles (Shingrix^®^; GSK plc, London, UK [21]) [14,22]. The unique architecture of liposomes allows the entrapment of dissolved hydrophobic solute in the inner core or encapsulation of hydrophobic components within the lipidic bilayer. The latter allows the use of an antigen-bilayer anchoring strategy for greater encapsulation and loading efficiency compared to simple antigen encapsulation. Conjugating a hydrophilic peptide antigen to a hydrophobic moiety, anchors the conjugates to the liposome membrane, exposing the antigen to both the interior and exterior of the liposomal surface. Furthermore, the incorporation of self-adjuvanting amphiphilic peptide vaccine constructs (e.g., lipopeptide and polymer-peptide conjugates) into liposome delivery systems can generate long-lasting immunity [23,24,25,26,27].

**Figure 1:**
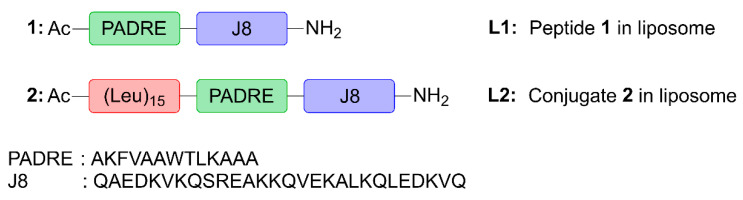
The structures of peptide **1** and polyleucine conjugate **2**, which were encapsulated into liposome delivery systems, **L1** and **L2**.

Here, we assessed the ability of poly(hydrophobic amino acid) (pHAA), a potent self-adjuvanting, hydrophobic polymeric chain of amino acids [28,29], to be anchored to liposomes. Compound **2**, a lead pHAA-antigen conjugate from prior investigations [29], was chosen for its high immunostimulatory activity in peptide-based vaccines against Group A Streptococcus (GAS). This vaccine candidate (Figure 1) was designed by conjugating a polyleucine (15-mer) to peptide **1** containing pan human leukocyte antigen-antigen D-related (HLA-DR) binding epitope (PADRE; sequence: AKFVAAWTLKAAA) universal Th-cell epitope, and J8-conserved B-cell epitope (derived from GAS M protein; sequence: QAEDKVKQSREAKKQVEKALKQLEDKVQ), to create polyleucine conjugate **2**. This was subsequently incorporated into cationic liposomes (**L2**). Peptide **1**, polyleucine conjugate **2** and their liposomal formulations (**L1** and **L2**, respectively) were tested for their ability to induce humoral immune responses after subcutaneous and intranasal immunization in mice.

## 2. Materials and Methods

### 2.1. Materials

Acquisitions of L-amino acids (Butyloxycarbonyl (Boc)-protected) were made from Mimotopes, Merck Chemicals (Darmstadt, Germany), and Novabiochem (Melbourne, Australia). HATU (1-[Bis(dimethylamino)methylene]-1H-1,2,3-triazolo [4,5-b]pyridinium 3-oxid hexafluorophosphate) was brought from Mimotopes. The following substances were purchased from Merck: acetonitrile, dichloromethane (DCM), methanol, N,N-dimethylformamide (DMF), N,N-diisopropylethylamine (DIPEA), piperidine, trifluoroacetic acid (TFA), and phenylmethanesulfonyl fluoride (PMSF) (Hohenbrunn, Germany). Resin (rink amide p-methyl-benzhydrylamine hydrochloride (pMBHA • HCl); substitution: 0.59 mmol/g; 100–200 mesh) was obtained from Peptides International (Louisville, KN, USA). Phosphate-buffered saline (PBS) was obtained from eBioscience (San Diego, CA, USA). Alexa Fluor 594 anti-mouse CD11c was purchased from BioLegend (San Diego, CA, USA). C57BL/6 mice were purchased from Animal Resource Centre (ARC; Perth, Australia). GAS clinical isolates, D3840 (nasopharynx swab) and GC2 203 (wound swab), were provided by the Princess Alexandra Hospital (Brisbane, Australia). The following items were obtained from Thermo Scientific: RPMI 1640 medium, paraformaldehyde (PFA), 3-(4,5-dimethylthiazol-2-yl)-2,5-diphenyltetrazolium bromide (MTT), horse blood Todd-Hewitt broth (THB), Ammonium-Chloride-Potassium (ACK) lysis buffer, L-glutamine, HEPES, 2-mercaptoethanol (Victoria, Australia). Goat anti-mouse IgG horseradish peroxidase (IgG-HRP) secondary antibody was supplied by Millipore (Burlington, MA, USA). Complete freund’s adjuvant (CFA), and secondary antibodies of the goat anti-mouse IgA-HRP and goat anti-mouse IgG1-HRP, were procured from Invivogen (San Diego, CA, USA). Goat anti-mouse IgG2c HRP (IgG2c-HRP) secondary antibody was purchased from Abcam (Cambridge, UK). The human lung carcinoma (NCIH460) cell line was obtained from American Type Culture Collection (ATCC; Manassas, VA, USA). Analytical-grade Tween 20 was purchased from VWR International (Queensland, Australia). Cholesterol, didodecyldimethylammonium bromide (DDAB), and dipalmitoylphosphatidycholine (DPPC) were purchased from Avanti Polar Lipids (Alabaster, AL, USA). Human embryonic kidney (HEK293) and human colon adenocarcinoma (SW620) cell lines from American Type Culture Collection (ATCC) was obtained from Invitro technologies (Brisbane, Australia). Chloroform, dimethyl sulfoxide (DMSO), o-phenylenediamine dihydrochloride (OPD) substrate, pilocarpine hydrochloride, cholera toxin B subunit (CTB), 5(6)-carboxyfluorescein (FAM), bovine serum albumin (BSA) and all other reagents were purchased from Sigma-Aldrich (Victoria, Australia).

### 2.2. Synthesis of Compounds ***1*** and ***2***

Compounds **1** and **2** were synthesized with the help of microwave-assisted solid-phase peptide using an SPS CEM Discovery reactor (Matthews, NC, USA) at a 0.1 mmol scale using pMBHA • HCl resin with a loading capacity of 0.59 mmol/g (SPPS; 70 °C, 20 W), as previously described [29]. Boc deprotection was achieved using TFA at room temperature (RT) for 2 min (twice), followed by DMF washing. Amino acids (0.84 mmol/g, 4.2 equiv) were activated using coupling solution containing 0.5M HATU (1.6 mL, 4.0 equiv) as an activator, and DIPEA (0.18 mL, 5.2 equiv.) as the base. Amino acid double-coupling was performed at 70 °C for 5 and 10 min, respectively. Once synthesis was complete, the peptide was N-terminus acylated with acetylation solution (90% DMF, 5% DIPEA, and 5% acetic anhydride), then the resin was washed (with DMF, DCM and methanol) and dried. The peptide was cleaved from the resin, using anhydrous HF with p-cresol as a scavenger. The crude compound was precipitated by cold diethyl ether, collected by filtration, and redissolved solvent B (90% acetonitrile, 10% Milli-Q water, 0.1% TFA) and in solvent A (100% Milli-Q water, 0.1% TFA) at a ratio of 1:1 (compound **1**) or solvent B only (compound **2**), and lyophilized. Shimadzu preparative reverse-phase high-performance liquid chromatography (RP-HPLC; Kyoto, Japan; LC-20AP × 2, CBM-20A, SPD20A, FRC-10A; equipped with a Vydac protein C18 (218TP1022; 10 μm, 22 × 250 mm) or C4 (214TP1022; 10 μm, 22 × 250 mm) column) was used to purify the compounds with solvent B (specific gradients for each compound) for 25 min, followed by compound detection at 214 nm. The collected fractions containing purified peptide were pooled, freeze-dried, and analyzed using analytical RP-HPLC on Vydac column (C18 (218TP54; 5 μm, 4.6 × 250 mm) or C4 (214TP54; 5 μm, 4.6 × 250 mm), for 40 min with 0−100% gradient of solvent B, and compound detection at 214 nm). The compounds were further confirmed using electrospray ionization mass spectrometry (ESI-MS).

Compound **1** yield: 20%. Purity: 99%. Molecular weight: 4653.42 g/mol. ESI-MS: [M+3H]^3+^ *m*/*z* 1,552.7 (calcd: 1552.1), [M+4H]^4+^ *m*/*z* 1164.5 (calcd:1164.4), [M+5H]^5+^ *m*/*z* 932.1 (calcd: 931.7), [M+6H]^6+^ *m*/*z* 776.8 (calcd: 776.6), [M+7H]^7+^ *m*/*z* 666.0 (calcd: 665.8). t_R_: 25.0 min (0–100% solvent B, 40 min, C18 column).

Compound **2** yield: 25%. Purity: 98%. Molecular weight: 6,350.82 g/mol. ESI-MS: [M+4H]^4+^ *m*/*z* 1,588.3 (calcd: 1,588.7), [M+5H]^5+^ *m*/*z* 1272.0 (calcd: 1271.2), [M+6H]^6+^ *m*/*z* 1060.3 (calcd: 1059.5), [M+7H]^7+^ *m*/*z* 908.0 (calcd: 908.3), [M+8H]^8+^ *m*/*z* 794.0 (calcd: 794.9), [M+9H]^9+^ *m*/*z* 705.5 (calcd: 706.7). t_R_: 39.0 min (0–100% solvent B, 40 min, C4 column).

### 2.3. Nanoparticle Formation

Compounds **1** and **2** were individually dissolved in PBS at a concentration of 2 mg/mL. Each mixture was sonicated for 30 min.

### 2.4. Liposomal Vaccine Formulation

Compounds **1** and **2** were encapsulated into liposome delivery systems using film hydration to produce **L1** and **L2**, respectively. Individual stock lipid solutions containing DPPC (10 mg/mL), DDAB (10 mg/mL), and cholesterol (5 mg/mL) were prepared in chloroform. Compound **1** (2 mg) was dissolved in 1 mL of methanol and mixed with 0.5 mL of DPPC, 0.2 mL of DDAB, 0.2 mL of cholesterol, and an additional 2 mL of chloroform. The lipid films were formed by slowly removing all organic solvent in the mixture under reduced pressure on a rotary evaporator. The films were placed under vacuum overnight (>16 h) to continue the drying process. PBS (1 mL) was added gradually to rehydrate the dried thin lipid films. The liposome solution was then gently vortexed for 5 min, followed by water bath sonication for 10 min (1 min intervals), to facilitate the homogenization process. The size of the liposome solution was manipulated using either extrusion (21 times at 55 °C; Avanti Polar Lipids, Alabaster, AL, USA; 100 nm polycarbonate membrane) or probe sonication (Model 3000; BioLogics, San Francisco, CA, USA; 40% power and 50% pulse for 5 min; repeated 3 times) to produce small-sized liposomes, **L1**. The extruded liposomes were compared with sonicated liposomes to discern differences between the size manipulation methods. Liposome **L2** was formulated using compound **2**, in the same manner as **L1**.

### 2.5. Physiochemical Characterization and Stability Analysis

Individual compounds, **1** and **2** (2 mg/mL in PBS), and liposomes, **L1** and **L2** (both extruded and sonicated; 1:10 diluted to 0.2 mg/mL of antigen in PBS), were analyzed by dynamic light scattering (DLS) to measure particle size (zeta intensity), size distribution (polydispersity index (PDI)), and charge (zeta potential) and size stability (after 7 days at RT). The samples (*n* = 5) were transferred into disposable DTS1070 folded capillary cells (Malvern Panalytical, Malvern, UK) before measurement at 25 °C and 173° light scattering.

The morphology of nanoparticles (1:2 dilution of samples prepared for DLS analysis) and liposomes (as prepared for DLS analysis) was captured via JEM-1010 transmission electron microscopy (TEM; HT7700 Exalens, HITACHI Ltd., JEOL Ltd., Tokyo, Japan), operated at 80 kV. The samples were applied to mesh grids and negative stained with 1% uranyl acetate (liposomes) or 2% phosphotungstic acid (nanoparticles) prior to imaging.

The stability of the nanoparticles (**1** and **2**) and liposomes (**L1** and **L2**) in a biological milieu was assessed by incubating samples with FBS [30]. Nanoparticles and liposomes were incubated with FBS at a concentration of 200 μg/mL at 37 °C for 1 h prior to analysis. The volume ratio of nanoparticle or liposome solution to 100% FBS was 1:9. Nanoparticle or liposome were prepared in 1:9 volume ratio to 100% FBS. The size and morphology of the samples were analyzed using DLS and TEM, as described above.

### 2.6. Liposomal Entrapment Efficiency

The entrapment efficacy of compounds **1** and **2** in liposomes (**L1** and **L2**, respectively) was determined by ultracentrifugation using an Optima TLX ultra-centrifuge (Beckman, CA, USA; TLA 120.2 rotor). Liposome samples (diluted to 0.25 mg/mL antigen concentration; 1:4 dilution) were centrifuged at 80,000 rpm (279,000× *g*) for 2 h at 4 °C to separate non-entrapped compounds from the liposomes and their contents. A 50 µL aliquot of the supernatant (containing free compounds) was further diluted with 50 µL of methanol (1:2 dilution) to fully dissolve the compounds. The mixtures (30 µL) were injected into analytical RP-HPLC (on C4 and C18 Vydac columns for **L2** and **L1** samples, respectively) using a solvent B (0–100% gradient) for 30 min and the compound was detected at 214 nm. The area under the compound peak was used to calculate the concentration of free compounds, in reference to the concentration–absorbance standard curve (Supplementary Material, Figure S1) of individual compounds. The entrapment efficiencies (%) of the liposomes were calculated as:

$$\frac{\left(1\;\mathrm{mg}/\mathrm{mL}-\left(\mathrm{Concentration}\;\mathrm{of}\;\mathrm{free}\;\mathrm{compound}\;\left(\mathrm{mg}/\mathrm{mL}\right)\;\times \;4\;\times \;2\right)\right)}{1\;\mathrm{mg}/\mathrm{mL}}\times \;100\%\;.$$

### 2.7. MTT Cytotoxicity Assay

Cytotoxicity evaluation of compound **2** and liposome **L2** was performed via MTT assay on HEK293 and SW620 adherent cell lines, as reported previously [31]. These cells were seeded into a 96-well round bottom plate at a density of 5000 cells/well for HEK293 and 2000 cells/well for SW620 in 190 µL of culture medium (DMEM for HEK293 cells and RPMI 1640 for SW620) supplemented with 2 mM L-glutamine, 100 µg/mL streptomycin, 100 U/mL penicillin, and 10% FBS. The plates were incubated at 37 °C for 24 h with 5% CO_2_ to allow cell attachment. The cells were treated with 10 µL/well of compound **2** and liposome **L2** (at concentrations of 0.25, 0.5, 1, and 2 mg/mL, each). Sodium dodecyl sulfate (SDS, 20% *w*/*v* in water) was used as a positive control, while untreated cells were used as negative controls. All experiments were conducted in duplicate. After incubation, the solution was replaced with 20 µL of MTT reagent (5 mg/mL in PBS) and 100 µL of fresh culture medium and incubated with 5% CO_2_ for 4 h at 37 °C. The culture medium was then aspirated to form formazan crystals, which were solubilized with 100 μL/well of DMSO and incubated for 30 min at 37 °C with 5% CO_2_. The absorbance of each well was read at 580 nm.

### 2.8. In Vitro Uptake

Bone marrow-derived dendritic cells (BMDCs) were extracted from mice (6–8-week-old C57BL/6). C57BL/6 mice were culled by CO2 asphyxiation; immediately after, the tibia and femur were extracted and soaked in ethanol (70% *v*/*v*) for 1 min. The tibia and femur of each mouse were washed and then collected separately in a fresh tube, which contained sterile RPMI (supplemented with, 2 mM L-glutamine, 100 units/mL penicillin, 100 μg of streptomycin; 20 mM HEPES, 0.1 mM 2-mercaptoethanol, and 10% FBS complete media (CM)). The bones were then flushed after top ends of each bone were cut off to facilitate the BMDC extraction. Cells were then dissociated with a pipette (1 mL), filtered through a 100 μm cell strainer (Millipore, Burlington, MA, USA) into a centrifuge tube (10 mL) and centrifuged for 5 min at 1400 rpm at RT. Then, the supernatant was removed, and cells were re-suspended in ACK lysis buffer (1 mL) and incubated for 1 min. The lysis buffer reaction was stopped by adding 9 mL of CM and cells were centrifuged again at 1400 rpm for 5 min at RT. Again, the supernatant was removed carefully, and the BMDCs were re-suspended in 4 mL of CM and counted. Microscope cover slips were soaked in 70% *v*/*v* ethanol and placed into each well. The BM cells concentration in CM was then adjusted to 5 × 10^5^ cells/mL and plated in 6-well plates (Corning; Glendale, AZ, USA). To the cell suspension GM-CSF (PeproTech, Rocky Hill, NJ, USA) was added at a final concentration of 10 ng/mL. BM cells concentration was adjusted to 5 × 10^5^ cells/mL in CM and incubated at 37 °C in 5% CO_2_ for 3 days. On day 3, these cells were incubated with FAM-conjugated compound **2** or liposome **L2** for 8 h.

After 8 h, the cells were prepared for cell surface staining by washing (twice) with 1 mL of PBS. After PBS aspiration, the cells were fixed with 1 mL of 4% PFA (20 min at RT), washed, and blocked using 1% BSA (1 h at RT). Cells were then washed and incubated with CD11c at 1:200 dilution for 1 h at room temperature. Cells were then washed, before the microscope slides extracted from the wells, dried, and mounted with DAPI mounting medium and a cover slip.

Microscopy images were obtained with a Nikon 90i Microscope at 60x in oil immersion with Widefield Fluorescence on Laser scan Confocal modality, Pinhole Size 30.0 µm and captured on 3 different channels (Dapi, Alx594 and FITC) on BRG Confocal mode (Laser Wavelengths 408.0, 488.0, 561.0) and captured with NIS elements software AR 4.13.5. Images were subsequently annotated with scalebar using ImageJ (version 1.51).

### 2.9. In Vivo Mouse Vaccination

Six-week-old C57BL/6 female mice were housed at the AIBN Animal Facility at The University of Queensland; they were acclimatized before experimentation. Mice were immunized (subcutaneously or intranasally) with 30 µL of compound **2**, or liposomes **L1** or **L2** (each containing 60 µg of antigen) on day 0, followed by three boosts of the same dose on days 21, 28, and 35 (Supplementary Material, Figure S2). Positive control mice were given 60 µg of peptide **1** mixed with CFA (1:1 volume ratio) or CTB (10 µg) for subcutaneous and intranasal immunization, respectively. Negative control mice received 30 µL of PBS.

### 2.10. Collection of Serum and Saliva

Blood from individual mice were collected on days −1 (naïve serum), and 20, 27, 34, and 49 after primary immunization and processed to retrieved serum samples for J8-specific IgG antibody analysis (Supplementary Material, Figure S2). Tail tip blood (10 µL diluted in 90 µL of PBS on days 20, 27 and 34) and heart puncture blood (1 mL blood collected on day 49) were centrifuged at 3600 rpm (956× *g*) for 10 min, to collect the serum from the supernatant.

Saliva samples were also collected (on days −1 (naive saliva) and 42 after primary immunization) for J8-specific IgA and IgG analysis. A total of 50 µL of 0.1% pilocarpine was intraperitoneally injected, to stimulate salivation. A protease inhibitor (2 µL in 100 mM PMSF) was added to the collected saliva samples (100 µL).

Serum and saliva samples were kept at −80 °C until further analysis.

### 2.11. Detection of Antivody Titer

Enzyme-linked immunosorbent assays (ELISA) were used to quantify J8-specific antibody (IgG, IgG1, IgG2c, and IgA) titers. J8 antigen (50 µg) was coated onto a 96-well plate. Serial dilutions (two-fold) of the samples were carried out, starting with a 1:100 concentration of serum and a 1:4 concentration of saliva. Both saliva and naive mouse sera were included as the negative controls. The samples were incubated for 1 h at 37 °C and then washed. The plate was treated with a secondary antibody (diluted 1:3000 IgG-HRP for serum and diluted 1:100 IgG- or IgA-HRP for saliva) for 1 h at 37 °C. The plates were washed incubated with OPD substrate for 20 min at RT. The plate content absorbance was read at 450 nm using a SpectraMax microplate reader (Molecular Devices, Silicon Valley, CA, USA). The antibodies titers were presented as the lowest feasible dilution with absorbance greater than 3× the standard deviation above the control wells mean absorbance.

### 2.12. Antibody Opsonization Assays

Opsonization assays were carried out on heat-inactivated day-49 serum samples (50 °C for 30 min) using GC2 203 and D3840 GAS clinical isolates, as previously described [29]. Bacteria were prepared on THB agar plates (supplemented with 5% yeast extract) and incubated (37 °C, 24 h). A single colony from each bacterium was replicated to approximately 4.6 × 10^6^ CFU/mL in THB solution (supplemented with 5% yeast extract). The bacteria cultures were two-fold serially diluted with PBS in a 96-well plate. Then, 10 μL of heat-inactivated sera and 80 μL of horse blood were added. Assays with three different separate cultures were performed in triplicate. Serum-containing bacteria were incubated for 3 h at 37 °C. The solution (10 μL) was plated on supplemented THB agar plates and incubated for 24 h at 37 °C. The CFU enumerated from the plates was used to measure bacterial survival rate.

### 2.13. Ethics Statement

This study was performed according to strict regulations set by the National Health and Medical Research Council (NHMRC) of Australia (Australian Code of Practice for the Care and Use of Animals for Scientific Purposes, 8th edition, 2013). All animal procedures and protocols were approved by The University of Queensland Animal Ethics Committee (AEC Approval Number: SCMB/AIBN/069/17) or the of Royal Melbourne Institute of Technology (RMIT) University Research Animal Facility (RAF; AEC Approval Number: 1917).

## 3. Results

### 3.1. Preparation and Characterization of Vaccine Candidates

Following the previously published vaccine design [29], linear polyleucine conjugate **2** was synthesized by coupling 15 copies of leucine to the N-terminus of peptide **1** bearing PADRE and J8 epitope (Figure 1). This linear construct was more efficient in stimulating antibody production than the original branching arrangement [28,29]. Compounds **1** and **2** were synthesized via Boc-SPPS, purified using preparative RP-HPLC. Their structure and purity (>95%) were confirmed using ESI-MS and analytical RP-HPLC (Supplementary Material, Figure S3).

**Figure 2:**
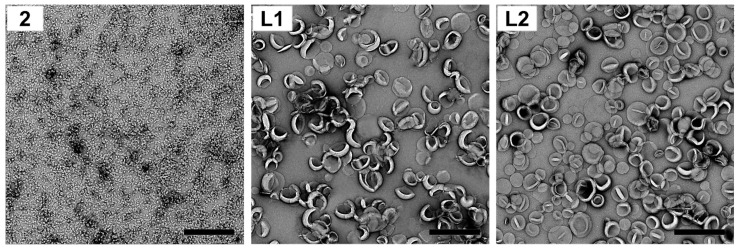
Particle images showing the morphology of compound **2** and encapsulated liposomes, liposomes **L1** and **L2**, captured by TEM (bar = 500 nm).

Polyleucine conjugate **2** was then self-assembled into nanoparticles in PBS, where it formed a milky suspension. Compounds **1** and **2** were also incorporated into cationic liposomes composed of DDAB:DPPC:cholesterol to produce cationic liposomes **L1** and **L2**, respectively. Uniform unilamellar vesicles were produced by extruding the liposomes through a 100 nm membrane. The particle size (zeta intensity), size distribution (PDI), charge (zeta potential) and stability of vaccine candidates **2**, liposomes **L1** and **L2** were determined using DLS (Table 1; see also Supplementary Material, Figures S2 and S3). TEM was used to visualize the liposomes, nanoparticles, and their aggregates (Table 1 and Figure 2). The entrapment efficiency of antigens (compounds **1** and **2**) in liposomes was also measured.

Linear polyleucine conjugate **2** self-assembled into small nanoparticles (~30 nm) and chain-like aggregates of nanoparticles (CLANs, ~170 and ~2400 nm) (Table 1 and Figure 2; see also Supplementary Material, Figure S4), similar to previous results [29]. When conjugate **2** was anchored to the liposome bilayer via its polyleucine tail, liposome **L2** showed a minor decrease in surface charge (19 mV) compared to the blank liposome (24 mV) and liposome **L1** (24 mV; Table 1). It is important to note that nanoparticles and liposomes formulated in PBS had reduced surface charge due to the adsorption of phosphate anions on the surface of cationic nanoparticles [32] and liposomes [33]. The presence of polyleucine in conjugate **2** improved antigen entrapment efficiency in liposomes in comparison to liposomal encapsulation of peptide **1** (liposome **L2** vs. liposome **L1**; 96% vs. 76%; Table 1). Liposomes bearing antigens (**L1** and **L2**)were similar in size to blank liposomes (130–140 nm; Table 1). All formulated liposomes had a uniform size distribution (PDI < 0.1, as measured by DLS; Table 1). Both liposomes **L1** and **L2** were stable at RT for at least 7 days (Supplementary Material, Figure S5) despite phosphate buffer’s ability to destabilize nanomaterials [32,33]. TEM images also revealed that liposomes **L1** and **L2** exhibited uniform and unilamellar liposome structures (Figure 2).

Nanoparticle and liposome stability was also investigated in the presence of fetal bovine serum (FBS). Compound **2** maintained its structure in serum (as measured by DLS and TEM; Supplementary Material,Figure S6). However, it was slowly released from the liposomes (**L2**) when incubated with serum, as shown by free small nanoparticles and CLANs with no liposome visible. A similar tendency was observed with liposome **L1**.

### 3.2. MTT Cytotoxicity Assay

The cytotoxicity of the lead vaccine candidates was examined using SW620 and HEK293 cell lines (Supplementary Material, Figure S7). Compound **2** did not impair the viability of human cell lines at doses of up to 2 mg/mL, indicating no pHAA-related toxicity. However, liposome **L2** showed slight toxicity with increased concentration, which is common for cationic liposomes: even those used in vaccine formulations [34,35].

### 3.3. In Vitro Uptake

Compound **2** and its liposomal formulation **L2** were tested for their affinity to BMDCs (Supplementary Material, Figure S8). The uptake study showed that they were both internalized by BMDCs cells, despite the differences in type of particle (CLAN nanoparticles **2** vs. liposome **L2**).

### 3.4. Immunological Evaluation of the Vaccine Candidate

**Figure 3:**
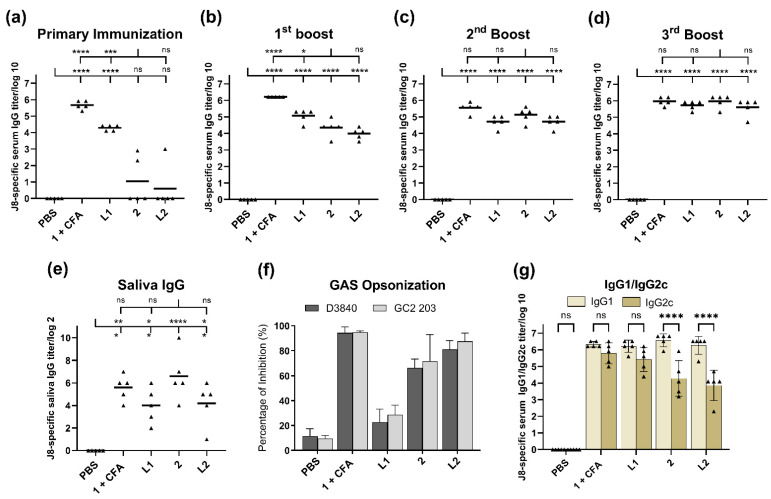
Immune responses after subcutaneous injection of PBS (negative control), compound **1** + CFA (positive control), compound **2**, and liposomes **L1** and **L2** in C57BL/6 mice (*n* = 5). J8-specific serum IgG antibody titers after (**a**) primary immunization, (**b**) first, (**c**) second, and (**d**) third boosts, (**e**) J8-specific saliva IgG antibody titers, and (**f**) J8-specific IgG1/IgG2c ratio in the serum collected in the final bleed, as analyzed by ELISA. Each point (triangle) represents an individual mouse; bars represent the average antigen-specific IgG antibody titers. Statistical analysis was performed using one-way ANOVA with Tukey’s multiple comparison test ((*) *p* < 0.05, (**) *p* < 0.01, (***) *p* < 0.001, (****) *p* < 0.0001). (**g**) Average opsonization percentage of different GAS strains (D3840 and GC2 203) by serum collected on day 49 following the final immunization of C57BL/6 mice (*n* = 5) with conjugate **2**, liposomes **L1** and **L2**, and controls. ns: not significant.

Immunological evaluation was performed in 6-week-old female C57BL/6 mice (Figure 3). The mice received four subcutaneous immunizations (primary immunization and three boosts) of peptide **1** + CFA (positive control), polyleucine conjugate **2**, or liposomes **L1** or **L2** (60 μg of compound **1** or **2** in each formulation) in 30 μL of PBS on days 0, 21, 28, and 35. The negative control group was treated with 30 μL of PBS.

All vaccine candidates induced significant J8-specific IgG titers (*p* < 0.0001) after the first boost (Figure 3b) compared to the negative control. Mice that received CFA-adjuvanted peptide **1** (positive control) produced the highest antibody titers. Following the second boost, conjugate **2**, liposomes **L1** and **L2** stimulated similar antibody levels to the positive control (Figure 3c,d). The vaccine candidates did not stimulate J8-specific IgA antibody production in the saliva, similar to other peptide vaccine candidates bearing PADRE Th-cell epitope [31,36]. Instead, we measured mucosal IgG production. The groups that received liposomal formulations had lower J8-specific saliva IgG titers than mice treated with **2** alone (Figure 3e); however, the differences were not significant. Peptide **1** encapsulated in liposomes (**L1**) induced significant production of serum IgG against GAS epitope after the primary immunization (Figure 3a); only minimal increases in antibody levels were observed after subsequent boosts (Figure 3b–d). Sera collected from mice immunized with liposome **L1** also showed poor opsonic activity, despite the high level of antibody titers induced (Figure 3g). On the other hand, compound **2** and liposome **L2** showed high opsonic capacity against the tested GAS strains. The balance of pro-inflammatory and anti-inflammatory responses was assessed by Th1/Th2 ratio (Figure 3f). The IgG2a gene was deleted in C57BL/6 mice [37]; therefore, IgG2c isotype level was measured instead. All vaccine candidates showed a higher production of J8-specific serum IgG1 titers compared to IgG2c, indicating a Th2-biased immune response.

## 4. Discussion

We investigated the potential of DDAB-based liposomal formulations for loading pHAA-antigen conjugate. Cationic liposomes are known for their ability to enhance immune responses, compared to negatively charged or neutral liposomes, due to their interaction with the anionic membrane of immune cells [35]. However, the production of stable liposomes was not possible with only cationic lipids and, therefore, neutral lipid DPPC and cholesterol were included to stabilize the liposomes. The potential of DDAB:DPPC:cholesterol liposomes as a vaccine delivery platform for GAS antigens has been recognized previously [31,38,39,40,41].

Polyleucine closely resembles natural transmembrane domains (of proteins), which are often leucine-rich (e.g., fibronectin leucine-rich transmembrane protein (FLRT) [42] and small leucine-rich proteoglycans (SLRP) [43]. We, therefore, hypothesized that polyleucine could be utilized as a liposomal surface anchoring moiety. Indeed, conjugate **2** was anchored to the liposomes, allowing almost complete antigen entrapment efficacy (96%; Table 1). Blank liposome, liposomes **L1** and **L2** were formulated using the lipid hydration method, with two methods applied to control liposome size: sonification and extrusion. However, sonication produced liposomes that were neither uniform (presence of micrometer-sized liposomes: 80–120 and 4000–5000 nm, and high size distributions: PDI = 0.3–1.0), nor stable (Supplementary Material, Figure S5). Therefore, extruded liposomes were selected for further study. The liposomes produced, **L1** and **L2**, were of similar size, charge, polydispersity and stability, despite the antigen being encapsulated in liposome **L1** and anchored in the bilayer of liposome **L2** (Table 1 and Figure 2; see also Supplementary Material, Figures S2 and S3).

In order to gain greater insight into nanoparticle stability in blood, the self-assembled nanoparticles and liposomes were assessed in serum, where major proteins (R-, β-, and γ-globulins and albumin) have the potential to destabilize nanoparticles. R- and β-globulins were identified as the major influencers in the release of hydrophobic molecules from polymeric nanoparticles compared to the other blood components (γ-globulins, albumin, and red blood cells) [30]. Interestingly, compound **2** was able to maintain its nanoparticle formation in the presence of these proteins (Supplementary Material, Figure S6). The stability and biocompatibility demonstrated suggest that conjugate **2** may be stable in biological milieus, which is critical for in vivo delivery applications. In contrast, liposomes **L1** and **L2** were not stable in serum and were observed to release the compounds from the lipid vesicles. Nevertheless, the compounds’ ability to self-assemble remained, even when released from the liposomes (Supplementary Material, Figure S6); therefore, this had minimal to no effect on the immune response (Figure 3).

Regarding the subcutaneous injection of conjugate **2**, liposomes **L1** and **L2** triggered high levels of J8-specific IgG titers after the second boost, comparable to those induced by CFA-adjuvanted peptide **1** (Figure 3c): the third boost was not needed to generate strong immune responses (Figure 3d). The triggered immune responses were rather balanced (based on Th1/Th2 ratio); however, conjugate **2** and liposome **L2** triggered a weaker IgG2c (the most potent pro-inflammatory and cytotoxic IgG subclass) [44] production compared to adjuvanted peptide **1** and liposome **L1** (Figure 3f). Excessive pro-inflammatory responses can be detrimental to a host as they can lead to uncontrolled tissue damage [45].

There was no significant difference in immune responses (serum IgG, IgG1 and IgG2c, saliva IgG, and GAS opsonization) stimulated by liposomes bearing compound **2** (**L2**) and compound **2** alone (Figure 3). This comparable level of responses could be attributed to the ability of compound **2** and liposome **L2** that were able to be taken efficiently by DCs (Supplementary Material, Figure S8). The capacity of DCs to identify and entrap antigens play a crucial role in adjuvant/antigen recognition as well as the activation of innate and adaptive immune responses. Interestingly, when liposomes of the same composition were used to anchor the lipopeptide-based vaccine against GAS, they generated much stronger immune responses than lipopeptides alone [38]. However, when alone, lipopeptides self-assembled into large aggregates, which may explain their weaker performance when not formulated into liposomes. The lower immune stimulating efficacy of larger particles is well-known [3,39,46,47]. In contrast, nanoparticles formed by conjugate **2** were sufficient for immune stimulation (Figure 3), rendering the addition of liposomes unnecessary. In contrast to the liposomal formulation, polyleucine conjugate **2** showed no sign of toxicity (Supplementary Material, Figure S7).

Encapsulation of peptide **1** into liposomes generated significant immune responses, even after single immunization in mice (Figure 3a). However, additional immunizations greatly enhanced antibody titers in mice treated with conjugate **2** and liposome **L2,** but not liposome **L1.** Moreover, liposome **L1** was unable to opsonize GAS (Figure 3g), most likely due to improper conformation of peptide **1** (adopted random coil conformation instead of helical, as per the native protein) [29]. This clearly demonstrated that while liposomes may enhance a vaccine’s ability to produce high antibody titers, this was not necessarily reflected in the quality of antibodies produced.

We also evaluated polyleucine conjugate **2**, and liposomes **L1** and **L2**, as mucosal vaccine candidates. No J8-specific antibody responses were induced following the intranasal immunization of mice (data not shown). This may be related to the significantly lower surface charge and lower mucoadhesive ability of our vaccine candidates formulated in PBS, as water-based formulations with more positively charged particles were often used as effective intranasal vaccine candidates. Overall, when delivered subcutaneously, polyleucine-conjugated antigenic peptide **2** elicited strong immune responses in mice, even without the help of liposomes.

## 5. Conclusions

In this study, we investigated the use of polyleucine conjugate with cationic liposomes as vaccine candidates against GAS. We demonstrated the potential of pHAA-liposome delivery systems, as ours induced high antibody titers that were opsonic against GAS clinical isolates. Despite the polyleucine unit being very efficient at anchoring liposomes, the cationic liposomal formulation did not improve the immunogenicity of the lead vaccine candidate. These findings further prove the capability of polyleucine as a potent vaccine delivery system for peptide antigens.

## Figures and Tables

**Table 1:** Physicochemical characterization of compounds **1**, **2**, and encapsulated liposomes, **L1** and **L2**.

Vaccine Construct	Particle Size (nm)		PDI	Zeta Potential (mV)	Entrapment Efficiency
	TEM	DLS *			
**2**	10–30 nm NP and CLANs	30 ± 1 168 ± 10 2381 ± 429	0.44 ± 0.02	3 ± 1	-
**L1**	Unilamellar liposome	134 ± 1	0.03 ± 0.02	24 ± 3	76%
**L2**	Unilamellar liposome	129 ± 1	0.05 ± 0.02	19 ± 1	95%
Blank Liposome	Unilamellar liposome	144 ± 1	0.02 ± 0.01	24 ± 2	-

* Size, as measured by intensity. NP = nanoparticles; CLANs = chain-like aggregates of nanoparticles.

## Data Availability

Not applicable.

## Supplementary Materials

The following supporting information can be downloaded at: https://www.mdpi.com/article/10.3390/vaccines10081212/s1, Figure S1: Standard curve of compounds **1** and **2**; Figure S2: Immunization schedule; Figure S3: Analysis of the purified compounds by analytical RP-HPLC and ESI-MS; Figure S4: DLS spectra of particle size; Figure S5: Liposome size stability; Figure S6: Vaccine candidate stability in serum; Figure S7: MTT cytotoxicity assays; Figure S8: Dendritic cell uptake.
