# Supplementary material for: Poly(hydrophobic Amino Acids) and Liposomes for Delivery of Vaccine against Group A Streptococcus
Source: Vaccines (Basel). 2022 Jul 29;10(8):1212. doi: 10.3390/vaccines10081212 (PMC9413763; doi:10.3390/vaccines10081212)
Supplement: Supplementary file 1 [file vaccines-10-01212-s001.zip › vaccines-1818164-supplementary.pdf]

Supplementary Materials

Poly(hydrophobic amino acids) and liposomes for delivery of vaccine against Group A Streptococcus

Armira Azuar[1], Harrison Y. R. Madge[1], Jennifer C. Boer[2], Jazmina G. Cruz[3], Jingwen Wang[1], Zeinab G. Khalil[4], Cyril Deceneux[2], Georgia Goodchild[2], Jieru Yang[1], Prashamsa Koirala[1], Waleed M. Hussein[1], Robert J. Capon[4], Magdalena Plebanski[2], Istvan Toth[1,4,5]* and Mariusz Skwarczynski[1,*]

[1] School of Chemistry and Molecular Biosciences, The University of Queensland, St. Lucia, QLD 4072, Australia
[2] School of Health and Biomedical Sciences, RMIT University, Bundoora West, VIC 3083, Australia
[3] Diamantina Institute, Faculty of Medicine, The University of Queensland, Woolloongabba, QLD 4102, Australia
[4] Institute of Molecular Bioscience, The University of Queensland, St. Lucia, QLD 4072, Australia
[5] School of Pharmacy, The University of Queensland, Woolloongabba, QLD 4102, Australia
* Correspondence: Email: i.toth@uq.edu.au; Tel: (617) 3346 9892 (I.T.) and Email: m.skwarczynski@uq.edu.au; Tel: (617) 3346 9894 (M.S.)

Supporting Information

Figure **S1**: Standard curve of compounds **1** and **2**;

Figure **S2**: Immunization schedule;

Figure **S3**: Analysis of the purified compounds by analytical RP-HPLC and ESI-MS;

Figure **S4**: DLS spectra of particle size;

Figure **S5**: Liposome size stability;

Figure **S6**: Vaccine candidate stability in serum;

Figure **S7**: MTT cytotoxicity assays;

Figure **S8**: Dendritic cell uptake;

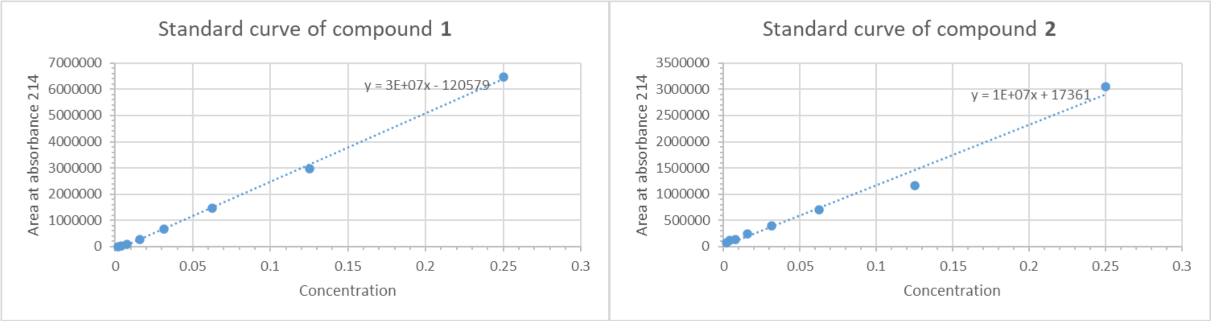

Figure S1. Standard curves of peptide **1** and polyleucine conjugate **2** used to calculate the concentrations of compound entrapped in the liposomes. These standard curves were plotted using area under compounds' peak against different compound concentrations (dissolved in PBS:methanol solution) that were injected (30 μL) into analytical RP-HPLC (on C18 or C4 Vydac column for compounds **1** and **2**, respectively) using 0-100% gradient of solvent B for 30 min with compound detection at 214 nm.

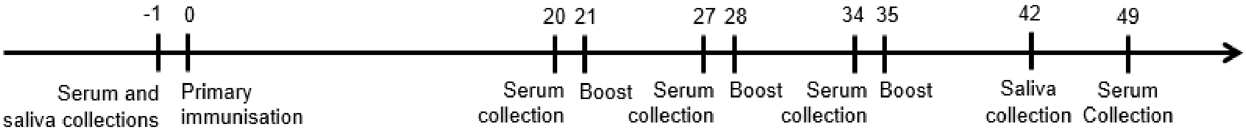

Figure S2. Immunization schedule with marked immunization, serum, and saliva collection days.

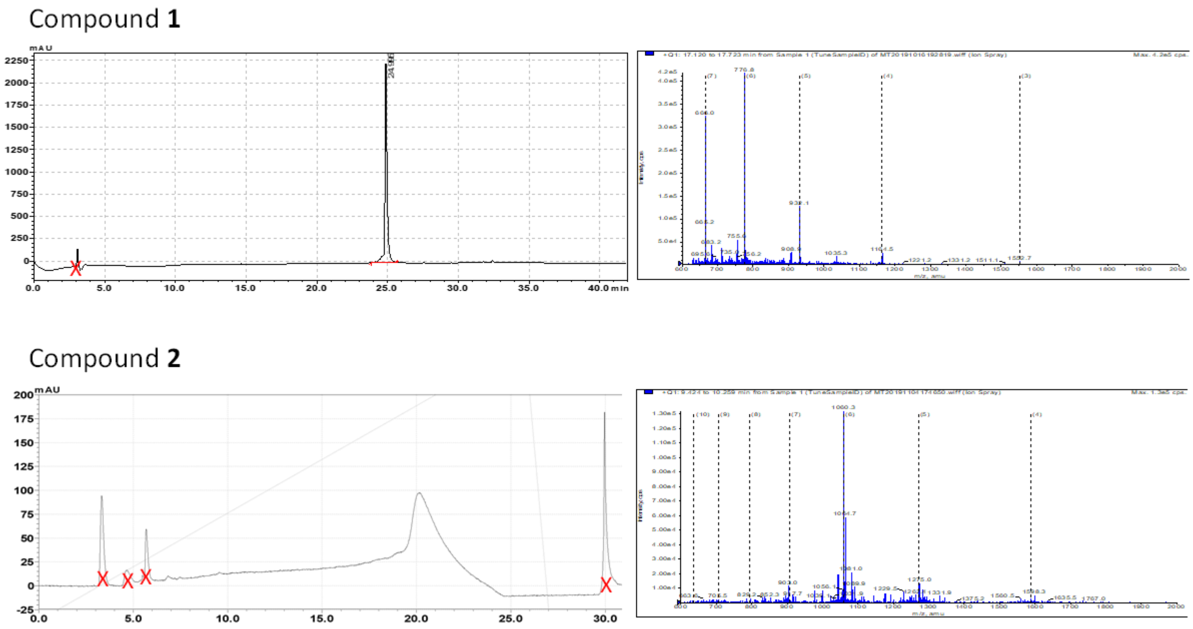

Figure S3. Analysis of purified compounds **1** and **2** by analytical RP-HPLC and ESI-MS. The compounds were purified using preparative RP-HPLC with solvent B concentration gradient 30-50% (**1**) and 65-85% (**2**) from Rt 5 min to 30 min. Analytical RP-HPLC graphs show pure compounds in single peak. The mass from these peaks, matched to the desired compounds in ESI-MS. "X" marks the background noise of the RP-HPLC column.

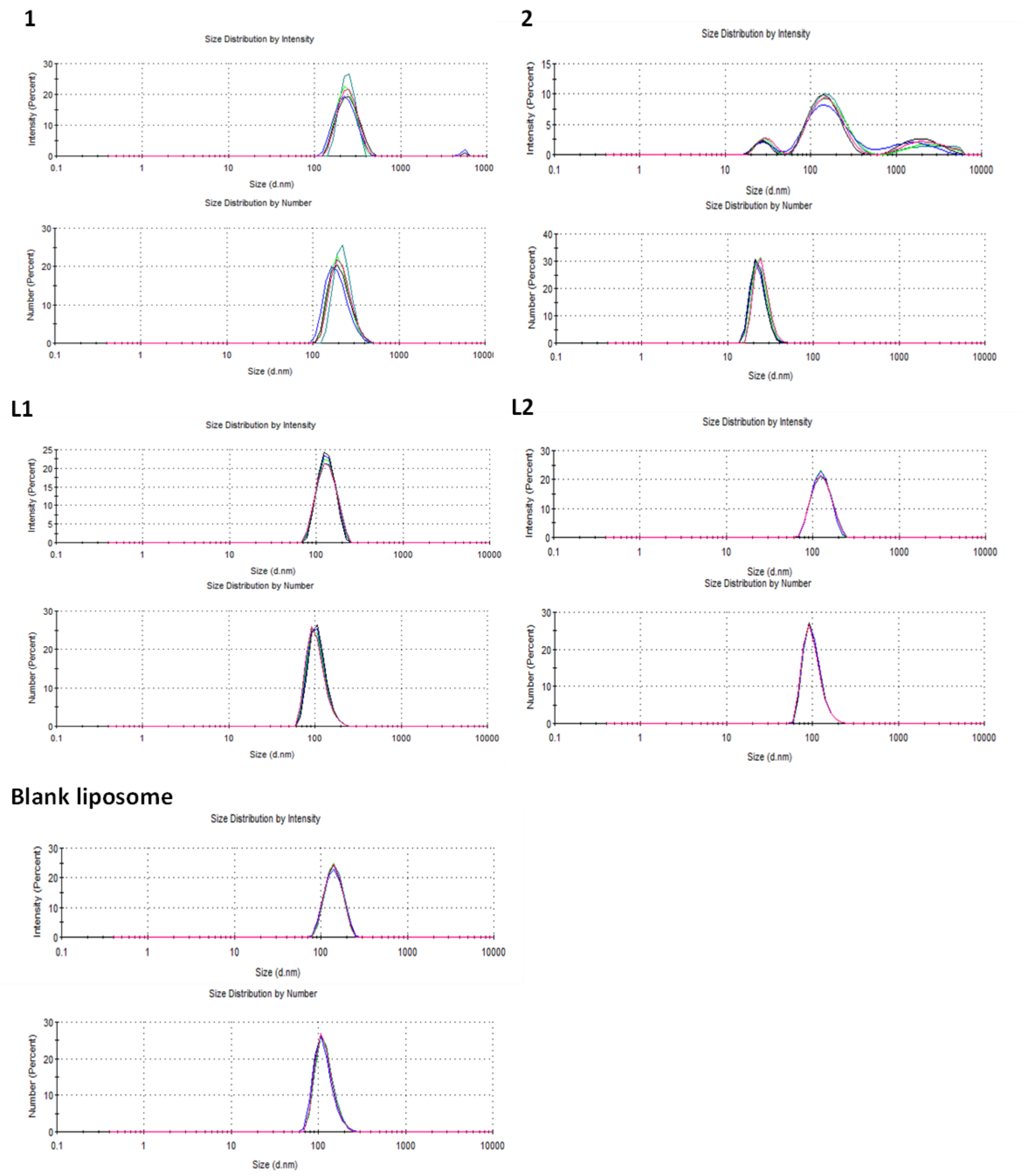

Figure S4. DLS spectra of particle size, by intensity (top) and number (bottom) for compounds **1** and **2**, encapsulated liposomes **L1** and **L2**, and blank liposome.

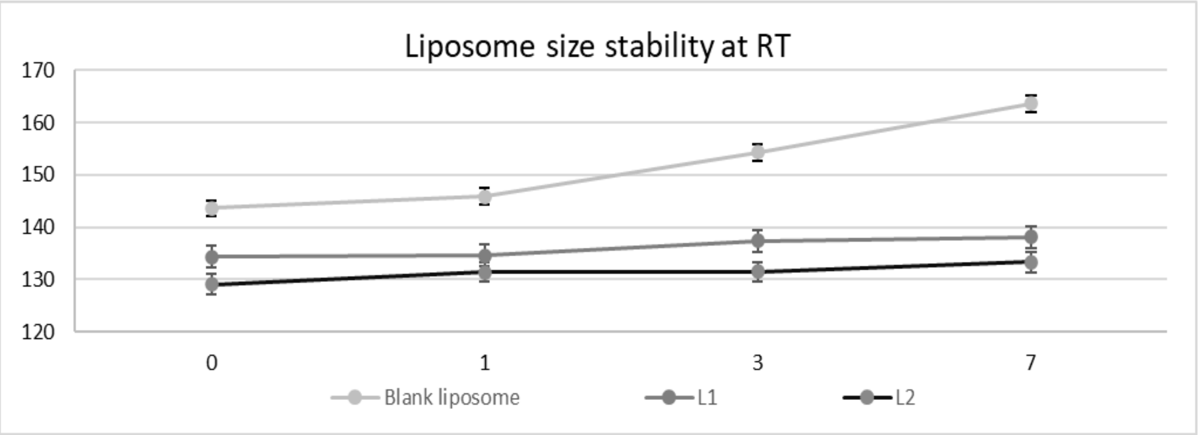

Liposome	Particle size (nm)		PDI	
	Day 0	Day 7	Day 0	Day 7
**Extrusion**				
**L1**	134 ± 1	138 ± 4	0.03 ± 0.02	0.02 ± 0.01
**L2**	129 ± 1	133 ± 3	0.05 ± 0.02	0.02 ± 0.01
**Blank Liposome**	144 ± 1	164 ± 2	0.02 ± 0.01	0.02 ± 0.01
**Sonication**				
**L1**	81 ± 1<br>4199 ± 536	88 ± 5<br>4514 ± 271	0.23 ± 0.02	1.00 ± 0.01
**L2**	121 ± 6<br>4737 ± 260	127 ± 5<br>4778 ± 163	0.19 ± 0.01	0.21 ± 0.02
**Blank Liposome**	79 ± 2<br>4469 ± 199	2690 ± 180	0.16 ± 0.01	0.26 ± 0.03

Figure S5. Liposome size stability over 7 days period at RT.

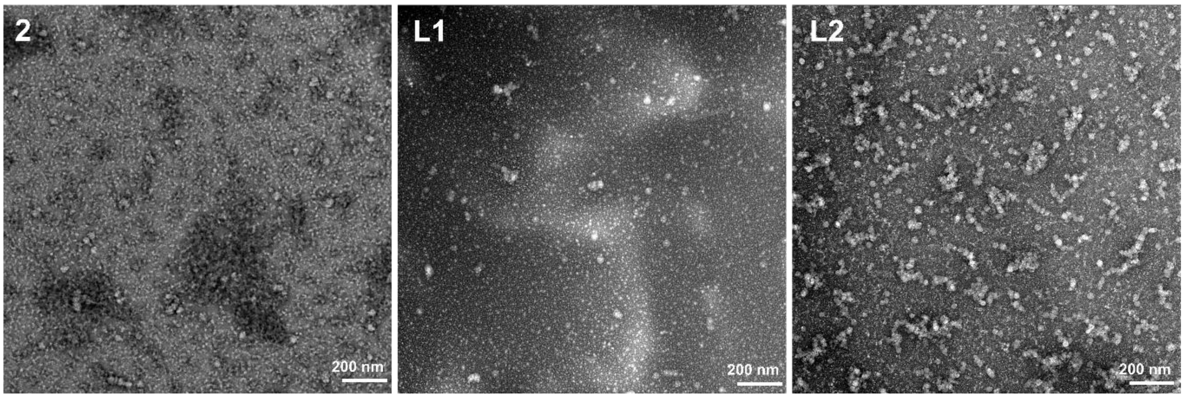

Compound	Particle size (nm)		PDI
	TEM	DLS[a)]	
**FBS**	Particles not visible (data not shown)	14 ± 1<br>59 ± 1	0.29 ± 0.01
**1**	Particles not visible (data not shown)	48 ± 1<br>4949 ± 282	0.31 ± 0.04
**2**	10-20 nm NP and CLANs	27 ± 1<br>153 ± 1<br>4393 ± 578	0.97 ± 0.05
**L1**	Liposomes and particles not visible	23 ± 5<br>164 ± 46<br>2244 ± 130	0.97 ± 0.05
**L2**	10-20 nm NP, CLANs, and 50-70 nm spherical aggregates	16 ± 2<br>47 ± 4<br>537 ± 24<br>4304 ± 530	0.99 ± 0.01

[a)]*size as measured by intensity.*
NP = nanoparticles; CLANs = chain like aggregates of nanoparticles

Figure S6. Stability of the vaccine candidates after incubation in FBS at 37°C for 1 hour.

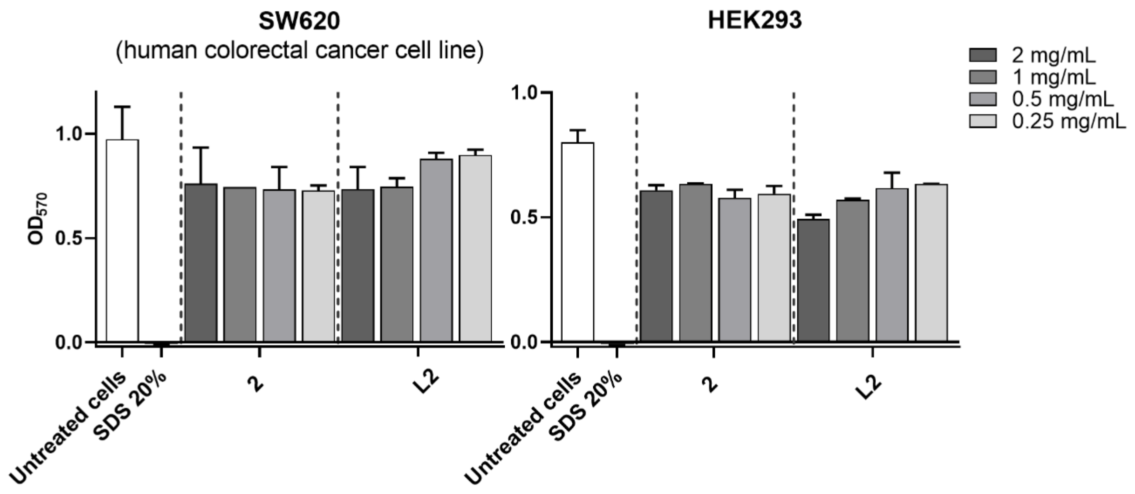

Figure S7. MTT cytotoxicity assay of polyleucine conjugate **2** and liposome **L2**.

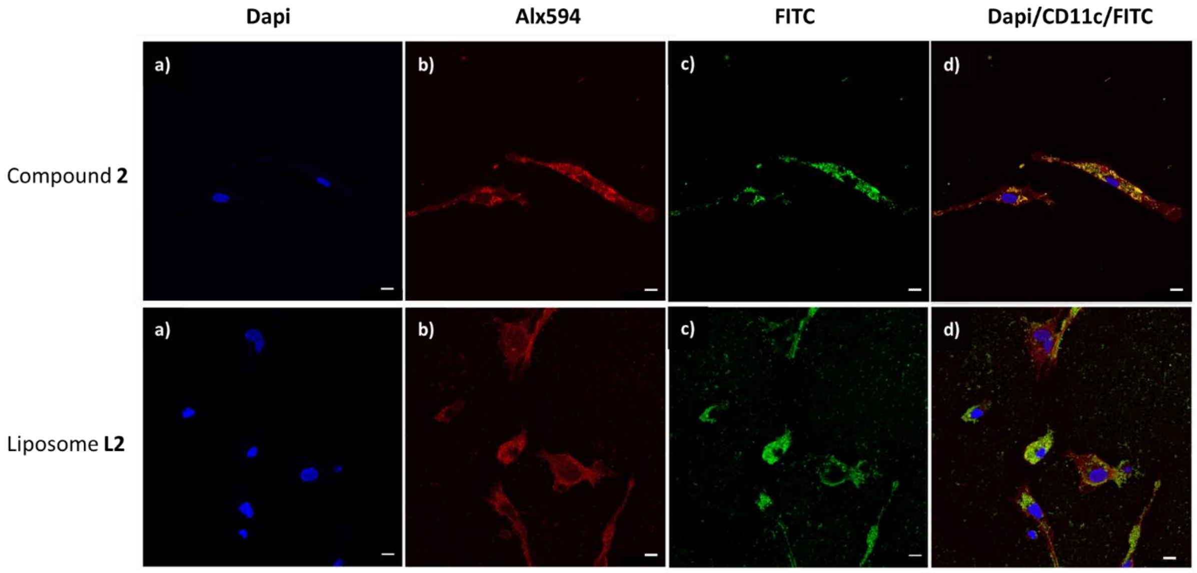

Figure S8. Microscopy of dendritic cells showing nanoparticle **2** and liposome **L2** uptake. Confocal 60x in oil immersion showing nanoparticle and liposome uptake. Cell channels were split to (**a**) Dapi channel showing cell nuclei (blue staining), (**b**) Alx594 channel showing CD11c cytoplasm (red staining; CD11c membrane marker was chosen to show nanoparticle/liposome uptake, where particles are surrounded by red staining in the membrane), (**c**) FITC channel showing nanoparticle or liposome (green staining) and (**d**) merged images showing nanoparticles or liposomes were uptaken into the cells.
